# Google Calendar: A single case experimental design study of a man with severe memory problems

**DOI:** 10.1080/09602011.2014.956764

**Published:** 2014-09-29

**Authors:** Victoria N. Baldwin, Theresa Powell

**Affiliations:** ^a^ School of Psychology, University of Birmingham, Birmingham, UK

**Keywords:** Google Calendar, Single case experimental design, Prospective memory, Acquired brain injury

## Abstract

A single case experimental design across behaviours was utilised to explore the effectiveness of Google Calendar text alerts delivered to a mobile phone as a memory aid. The participant was a 43-year-old man (JA) with severe memory problems and executive difficulties caused by a traumatic brain injury (TBI). JA was initially very unwilling to use any memory aid and so a detailed assessment of his beliefs about memory aids, his cognitive difficulties and his social context was performed and a set of specifications for an aid was produced collaboratively. Six weeks of baseline data and six weeks of intervention data were collected for three target memory behaviours and three control memory behaviours. Results were analysed using nonoverlap of all pairs (NAP) analysis which showed a reduction in forgetting in the three target behaviours and no change in two of the three control behaviours. A subjective measure (the revised Everyday Memory Questionnaire) also suggested improvement. This study illustrates that Google Calendar is a highly effective memory aid and emphasises the importance of choosing a memory aid to suit the person's lifestyle and beliefs.

## INTRODUCTION

The use of portable electronic aids that provide both a means of communication and continuous memory support throughout the day is now commonplace. Such aids are in keeping with current technological trends and are widely accepted. Devices include personal hand-held computers, e.g., mini notebooks and tablets, such as the iPad, mobile phones and smartphones. The present study describes the use of Google Calendar and a mobile phone as an electronic memory aid for a man with acquired brain injury (ABI) who found other memory strategies unacceptable on the basis that they were potentially stigmatising.

Memory impairment not only affects the ability to recall past information but also the ability to remember to perform intended tasks at specific times in the future, i.e., prospective memory (PM) (Fish, Wilson, & Manley, 2010). Everyday functioning depends heavily on PM and a deficit in this area is associated with increased disability and limited social participation, making it an important target for rehabilitation (Fleming, Shum, Strong, & Lightbody, 2005). Several studies of PM have shown that difficulties persist long after injury (e.g., Knight, Harnett, & Titov, 2005; Potvin, Rouleau, Audy, Charbonneau, & Giguere, 2011) and there is little evidence that suggests that lost memory functioning can be restored following ABI (Wilson et al., 2009).

In a review of the literature relating to PM functioning in closed head injury, Shum, Levin, and Chan (2011) identified seven studies using either a remedial/restoration or compensatory approach to treat PM impairments. Studies suggested that both approaches produced promising findings in terms of improvements in PM behaviour, although studies lacked long-term follow up. However, rehabilitation of memory functioning generally after ABI has tended to focus on compensatory approaches rather than techniques that aim to restore/retrain memory function. Cicerone et al. (2005) recommend that external compensatory strategies including assistive technology, should be a practice guideline in the treatment of people with moderate to severe memory problems and, in clinical practice, external aids have been reported to be the most widely used compensatory strategy (Evans, Wilson, Needham, & Brentall, 2003).

Paper-based aids, such as notebooks, calendars, lists and diaries, have been shown to be effective methods of compensating for memory difficulties and improving independence (e.g., McKerracher, Powell, & Oyebode, 2005; Sohlberg & Mateer, 1989). The disadvantage of paper-based aids is that they are passive reminders requiring individuals themselves to initiate using or checking them which, in itself, is a memory task (Wilson, Emslie, Quirk, & Evans, 1999). One way of overcoming this difficulty is through the use of electronic memory aids as they often include a cueing device that attracts the individual's attention to the task as well as having the facility to store information (Kapur, Glisky, & Wilson, 2004).

The most thoroughly investigated electronic aid for compensating for PM difficulties is NeuroPage, a portable pager that provides audio/vibration alerts (Hersch & Treadgold, 1994). Eight studies have explored the use of NeuroPage (Emslie, Wilson, Quirk, Evans, & Watson, 2007; Evans, Emslie, & Wilson, 1998; Fish, Manly, Emslie, Evans & Wilson, 2008; Wilson et al., 1999; 2009; Wilson, Emslie, Quirk, & Evans, 2001; Wilson, Emslie, Quirk, Evans, & Watson, 2005; Wilson, Evans, Emslie, & Malinek, 1997) and all have reported a significant improvement in achievement of target behaviours with NeuroPage relative to baseline. Neuropage also reduces the amount of prompting needed from carers and helps increase independence (Evans et al., 1998; Wilson et al., 1999). In a follow-up study, Martin-Saez, Deakins, Winson, Watson, and Wilson (2011) explored changes in the use of Neuropage 10 years after the original cohort study of 40 (Wilson et al., 2003). In the 2011 cohort, users were given the opportunity to use their mobile phone to receive messages and 17 of 40 chose to do so. The authors comment that for one person, using mobile alerts “normalised” the use of Neuropage and improved acceptance. The most frequent message sent each week remained similar to the original cohort, i.e., medication reminders. However, new uses emerged such as reminders relating to mood management. The authors also note that slightly fewer health authorities are referring to NeuroPage and one could speculate that this could be related to the set-up fee and rental costs. The fact that reminders are externally programmed means that Neuropage does not require a great deal of learning to be used effectively (Kapur et al., 2004), but it may not be a financially viable option for all.

Personal digital assistants (PDAs) are an alternative that involve a one off cost as they do not connect to a cellular network and so there is no rental plan. A number of studies have demonstrated the effectiveness of PDAs as memory aids for people with ABI (DePompei et al., 2008; Gentry, Wallace, Kvarfordt, & Lynch, 2008; Gillette & DePompei, 2008; Thone-Otto & Walther, 2003; Waldon, Grimson, Carton, & Blanco-Campal, 2012; Wright, Rogers, Hall, Wilson, Evans, & Emslie, 2001; Wright, Rogers, Hall, Wilson, Evans, Emslie, & Bartram, 2001) and there is evidence that participants continue to use PDAs for up to four years post-introduction (Kim, Burke, Dowds, Boone, & Park, 2000). However, cheaper models may have limited function whereas higher specification models have superfluous keys that can be confusing for people with ABI (Kapur et al., 2004). Furthermore, unlike mobile or smartphones, PDAs do not allow internet surfing or access to social media or mobile calls, amenities which are increasingly viewed as a conventional part of everyday life.

Scherer, Elias, and Weider (2010) note that the extent to which a device meets the user's personal everyday needs is important and Baldwin, Powell, and Lorenc (2011) further showed that people only wish to use aids with which they feel “comfortable” and which are consistent with their sense of identity. In fact in a follow on study, Baldwin (2012) found that “lifestyle fit” was a more important predictor of the use of memory compensations than factors such as mistaken beliefs about memory and injury-related factors. It has been proposed that some people may adopt coping strategies that reduce discrepancies and threats between pre-injury and current self or current self and hoped-for self (Gracey, Evans, & Malley, 2009) and indeed, Baldwin et al. (2011) found that a key factor leading to avoidance of memory aids was that they are an aversive reminder of the injury and a threat to pre-injury identity. Memory compensations should therefore reflect an individual's sense of self and lifestyle and be in keeping with their value systems. One way of addressing this issue is by using technology that is widely used and accepted by society and is an integral part of daily life, such as the mobile phone.

Nine previous studies have explored the use of mobile phones or smartphones as a memory aid following ABI (Culley & Evans, 2010; DePompie et al., 2008; Fish et al., 2007; Savage, & Svoboda, 2013; Stapleton, Adams, & Atterton, 2007; Svoboda & Richards, 2009; Svoboda, Richards, Leach, & Mertens, 2012; Svoboda, Richards, Polsinelli, & Guger, 2010; Wade & Troy, 2001). Two of the mobile phone studies used single case methodology. The first of these (Wade & Troy, 2001) consisted of a series of five single case AB designs in which pre-recorded spoken messages were sent to a standard mobile phone in people with moderate to severe memory problems. They found that the alerts were effective in increasing recall of target tasks for all five of their users. Stapleton et al. (2007) used a more rigorous design (ABAB) to examine the use of text message alerts in five people with memory problems caused by traumatic brain injury (TBI). Like Wade and Troy, they found benefits in two of their five participants who had mild to moderate memory difficulties but the three remaining participants who had more severe memory problems and executive difficulties did not benefit from the alerts. Wilson and Watson (1996) have also noted that for those with marked executive difficulties following ABI, the use of memory aids may not be as successful.

Two group studies have explored the broader application of mobile phone alerts as a means to facilitate goal-directed behaviour (Culley & Evans 2010; Fish et al., 2007). Text message alerts were sent to participants with memory problems as a reminder to carry out a mental review of their rehabilitation goals. This both increased success on a set task and also facilitated recall of goals.

The launch of the first iPhone in 2007 with its multiple functions and internet connectivity has opened up numerous affordable opportunities for memory support. Five studies have looked at the effectiveness of such smartphones as a memory aid (DePompie et al., 2008; Savage & Svoboda, 2013; Svoboda et al., 2010; 2012; Svoboda & Richards, 2009). DePompie et al. (2008) selected two people with memory and organisational problems who had used a hand-held PDA successfully in the past (one with TBI and one with intellectual disabilities). They were taught to use various functions on a smartphone including the calendar and encouraged to use the devices for other purposes, e.g., games. Although it is not possible to extract the smartphone results from other PDA results, the authors report that the phones increased organisational independence and they went on to develop a series of recommendations for teachers and clinicians who wish to introduce smartphones or PDA devices to students.

Svoboda and Richards (2009) and Savage and Svoboda (2013) demonstrated the successful introduction of a commercial smartphone to aid both prospective and retrospective memory difficulties in a woman (RR) with moderate to severe memory impairment. Not only did RR acquire the skills to use the smartphone calendar for targeted PM tasks, but she generalised the skill to prompt everyday activities and learned to use other applications, e.g., video games and to do lists. They supplemented their original ABAB design with an 18-month follow-up study and found that RR continued to use the smartphone with similar success rates to immediately after the initial intervention and at 4-month follow-up. However, completion of memory tasks reduced when an alternative smartphone (with no training) was used. They note that training in the use of the smartphone may generalise within brands but not across different brands. In a second single case ABAB design, Svoboda et al. (2010) demonstrated the benefits of a smartphone, in particular the calendar feature, for an 18-year-old woman with severe memory difficulties who had sustained her injury at the age of 13 years. They found that the rate of completing tasks (e.g., making phone calls at the correct time or attending social events) was significantly increased using the smartphone when compared to baseline. Furthermore, use of the smartphone reduced carer strain and improved her quality of life. Svoboda et al. (2012) went on to demonstrate the success of their training protocol in 10 individuals with memory problems of varying aetiology who were taught to use either PDAs or smartphones to cue a series of phone calls or everyday memory tasks. Significant improvement was found in day-to-day memory functioning for all 10 participants and, again, some individuals generalised the training to other software applications on the devices.

Thus, studies so far suggest that mobile and smartphones, in particular the use of text message alerts, are effective in people with memory problems. There are a variety of memory applications (apps) and software packages that can be used with smartphones, such as Google Calendar (used in the present study), Microsoft office calendar and Zoho Calendar. McDonald, Haslam, Yates, Gurr, Leeder, and Sayers (2011) also explored the use of Google Calendar in 12 people with ABI (TBI, stroke, anoxia, viral infection or heart attack). This was a randomised controlled crossover study comparing the effectiveness of Google Calendar with a standard paper diary. They found that both aids improved recall of prospective memory tasks, however, Google Calendar was found to be significantly more effective than the paper-based diary. Participants highlighted that the timed text message alerts were the most beneficial feature of the calendar system, as they provided a written visual prompt as well as the auditory alert. In the latter study participants had all used memory aids prior to the study. In the present study we explore whether Google Calendar is an effective memory aid for a man (JA) with severe verbal and visual memory difficulties and impaired executive functioning who did not use any memory aids prior to his injury and was initially very unwilling to use any aid subsequent to his injury. Google Calendar was eventually chosen over other forms of memory aid after a detailed assessment that included factors influencing acceptability to JA.

## METHOD

### Participant

JA was a 43-year-old Pakistani male who had lived in the UK since the age of 2 years. He was referred to our out-patient brain injury rehabilitation service following a TBI 6 months earlier as a result of an assault. JA had a Glasgow Coma Scale score of 4/15 on admission to hospital indicating that he had suffered a severe TBI. At the time of the study JA was living part-time in the family home with his wife and three children (visiting in the evenings) and part-time in a shared house with two other people (non-family members). JA had not been able to return to work following the incident, but this remained one of his goals.

JA complained of severe everyday memory problems including difficulties recalling people, events, where he had put things, conversations and appointments. He had missed important doctor and hospital appointments and constantly relied on his family to remember information for him. JA also had mild expressive dysphasia and had minor difficulties writing. Results of neuropsychological assessments (Table 1) show that JA had severe verbal and visual memory difficulties and reduced speed of information processing as well as impaired executive functioning. Given JA's education (JA attended school until the age of 16) and employment background (JA worked in a family run local convenience store) his premorbid IQ was considered to be within the average range.

**TABLE 1:** Results neuropsychological assessment

	*Test score*	*Percentile score*
WASI Verbal IQ	73*	4th
WASI Performance IQ	78*	7th
WASI Full Scale IQ	73*	4th
BMIPB List Learning A1–5	31	< 1st
BMIPB List Learning B	4	18th
BMIPB List Learning A6	3	< 1st
BMIPB Information Processing (adjusted score)	20	< 2nd
Rey Complex Figure Copy	32.5	16th
Rey Complex Figure Intermediate Recall	14	5th
Rey Complex Figure Delayed Recall	10	< 1st
Trails Test A (time)	59 seconds	10th
Trails Test B (time)	208 seconds	<1st
Hayling Sentence Completion Speed A	1*	<1st
Hayling Sentence Completion Speed B	1*	< 1st
Hayling Sentence Completion Accuracy	1*	< 1st
Hospital Anxiety and Depression Scale	16	
Beck Hopelessness Scale	9	

* Test Scaled score.

Notes: WASI = Wechsler Abbreviated Scale of Intelligence; BMIPB = Brain Injury Rehabilitation Trust (BIRT) Memory and Information Processing Battery.

Although showing insight and acknowledging his difficulties, JA was resistant to any memory compensation that he felt would expose his memory difficulty to others (e.g., a written diary, calendar or post-it notes). He held a very strong belief that people would think “less of him” if his difficulties were highlighted by using a memory aid. This was noted during the initial interview when JA expressed anxiety about the type of memory aid as well as worrying about forgetting information. JA demonstrated moderate to high levels of anxiety and depression as measured by the Hospital Anxiety and Depression Scale (Zigmond & Snaith, 1983) and the Beck Hopelessness Scale (Beck, Weissman, Lester, & Trexler, 1974). JA also completed two self-report questionnaires to supplement the interviews and provide additional outcomes. The Beliefs about Memory Aids Questionnaire (BMQ) was recently developed to elicit potentially unhelpful beliefs about using memory compensations (Baldwin, 2012). It consists of 32 items divided into five subscales (threat appraisals, lifestyle, inappropriate beliefs, personal control beliefs and treatment control beliefs). He also completed the Revised Everyday Memory Questionnaire (EMQ-r; Royle & Lincoln, 2008) in order to further clarify the extent of his memory difficulties. Both measures were repeated at the end of the study (week 13).

### Study design

Taking into consideration JA's beliefs it was important that the memory aid was deemed acceptable to JA and that the context for the study was as realistic and meaningful as possible in order to maintain his engagement. Thus, rather than creating activities to be remembered, the target activities were genuine tasks that were important to JA and his family. It was also anticipated that withdrawing the intervention might raise ethical issues and so it was decided to include control behaviours rather than an ABAB design. In order to ensure the study was as meaningful as possible for JA, six individual real-life behaviours or types of forgetting were identified. A discussion took place between JA, his wife and the researcher around which real-life behaviours or types of forgetting were causing the most disruption to their daily lives. JA identified that the three most important types of forgetting were forgetting appointments (including doctors, dentist and hospital), forgetting to attend the rehabilitation service, and forgetting to attend the mosque. These were therefore identified as target behaviours and had to be combined due to their individual infrequency to make one target event score. The remaining three types of forgetting were identified as control behaviours: losing keys, forgetting social events (e.g., going shopping, friends visiting), and forgetting to pass on messages to his partner. Although misplacing keys is essentially a retrospective memory task, it was felt that this would still give an indication of any potential spontaneous recovery in memory functioning. Passing on messages would have been amenable to the current intervention if a prompt had been delivered at a set time each day reminding him to reflect on whether anything had arisen that day that needed to be passed on.

All target behaviours were monitored by JA's partner, who kept a weekly record of instances of forgetting and/or instances of having to remind JA about a behaviour/event at a point where, had she not reminded him, an important task would have been forgotten. For example, if JA was not getting ready to attend the rehabilitation service as he was still wearing his pyjamas and he was due to be collected in 10 minutes, this would be recorded as an incidence of reminding.

When considering the use of electronic aids for individuals with cognitive difficulties, it is good practice to explore the client's level of insight and motivation, ensure that the device is tailored to the client's cognitive needs to facilitate ease of use, incorporate errorless learning techniques into training, and carry out training both at the rehabilitation centre and in the home environment (Gartland, 2004). These issues were therefore considered very carefully, as well as what type of information JA had difficulty remembering. Based on interviews with JA and his wife, six specifications for the memory aid were identified:

- JA must feel comfortable using the memory aid.
- It must be discrete and not an obvious memory aid.
- It must alert JA that something needed to be carried out, i.e., not just an alarm.
- It must be small and easily portable.
- There would be no hand-written elements within the device due to JA's adamant belief that writing things down on paper would not be discreet.
- It must have no additional costs.

The only memory aid that fulfilled all the criteria was Google Calendar (see below). Furthermore, JA had used a standard mobile phone and text messaging on a regular basis prior to his TBI and had continued to use it post-injury. JA did not wish to purchase a new phone and did not wish to utilise a smartphone, meaning that it was not possible to use an app that required internet access.

### Materials

Google Calendar (www.google.com/calendar) is a free online calendar provided by the search engine Google. It allows events to be entered for a specific time and date, synchronises with the user's mobile phone and sends SMS text alerts about the event, thus acting as a memory prompt. For each event added to the calendar, a maximum of five text alerts can be set. JA used his own mobile phone as he was familiar with this having used it for a number of years prior to his injury.

### Statistical analysis

The target and control behaviours specifically chosen by JA were genuine events and therefore differed in terms of weekly frequency. This meant that a percentage of events forgotten had to be calculated for each week. For target events forgotten, target events JA would have forgotten, and control social events, there were insufficient individual incidences of specific types of event in one week from which to derive a meaningful score. Therefore, types of events (e.g., going to the mosque, attending the rehabilitation centre and attending hospital appointments) were combined.

As well as visual inspection, statistical analysis of the data was also undertaken. Manolov, Solanas, Sierra, and Evans (2011) suggest that for data that is autocorrelated, nonoverlap of all pairs (NAP) analysis or slope and level change (SLC) analysis can be used. NAP is a method of measuring the degree to which data in one phase overlap with another phase (Parker & Vannest, 2009). It also has an advantage over parametric analysis (e.g., *t*-tests, ANOVA) because extreme outliers are common in single case research and parametric effect sizes are disproportionately influenced by them (Parker & Vannest, 2009). It can also be used when data does not meet the parametric assumptions of serial independence, normality and constant variance of residual scores (Parker & Vannest, 2009). NAP can be calculated using one of two methods: (1) calculate all nonoverlapping data (i.e., a nonoverlapping pair will have a phase B data point larger than its paired baseline phase A data point); (2) count all overlapping pairs and then subtract from the total possible pairs to obtain a nonoverlap count. Total possible pairs (total *N*) is the number of data points in phase A multiplied by phase B (N_A_ *N_B_) (Parker & Vannest, 2009).

### Procedure

Ethics approval was obtained from South Birmingham Research Ethics Committee and informed consent was obtained from both JA and his partner.

JA's partner recorded all target and non-target events that were forgotten as well as instances of reminding. She also received a text message reminder every evening to remind her to make the recording. Baseline data (phase A) were collected for six weeks. Week 7 was a training week during which JA was sent a series of text messages in order to familiarise him with the process of responding to the message alerts. He was asked to undertake a number of tasks including phoning the doctor's surgery to make an appointment, texting the researcher stating the time and date of the appointment, asking his key worker at the rehabilitation service for the envelope containing a form to complete, completing the form at home and returning it to his key worker. In fact JA did not require any prompting to respond to the text alerts and all tasks were completed successfully. Intervention data were then collected for a further six weeks (phase B) from week 8 to week 13. JA's partner was asked at the beginning of each week about any upcoming appointments or events, JA was also sent a daily text message asking him if any appointments or events needed to be put on the calendar. Although all events were entered onto Google Calendar by the researcher, JA stipulated how far in advance each text message alert was delivered. JA decided on five message alerts per event and indicated that for each event a message alert would be sent to him the night before at 10 p.m., then one message at 7 a.m. (which he would be able to look at when he woke up in the morning if later than 7 a.m.) followed by message alerts 1 hour, 30 minutes, 20 minutes and 5 minutes before the event.

However, if he was to use the aid independently once the study was complete, it was important to establish early on whether he would be able to enter events himself on his desk top computer at home. Thus, JA had weekly one and a half hour training sessions commencing in week 1 of the baseline phase. Training continued over a period of eight weeks and finished at the end of week 9 during the intervention phase. He was provided with a step-by-step guide with illustrated instructions on how to locate, enter, and navigate the calendar, input, edit or delete an event and how to set repeat reminder events. For each of these steps JA was first given a demonstration and then asked to carry out the step independently, initially using the written guide. In keeping with errorless learning principles (Baddeley & Wilson, 1994), JA was thus discouraged from guessing the next step and verbally prompted to refer to the guide. Only once he was confident that he could accurately execute a step was he allowed to disregard the manual. Each consecutive training session would begin with practice on the steps already learned using the manual if necessary, before going on to the next step. JA was eventually able to input events without verbal prompting and automatically referred back to the instruction manual if he needed reminding. However, JA only inputted events into Google Calendar for the purpose of the study during the training week (week 7), he did not input any events into the calendar himself for the remainder of the study.

## RESULTS

**Figure 1.**
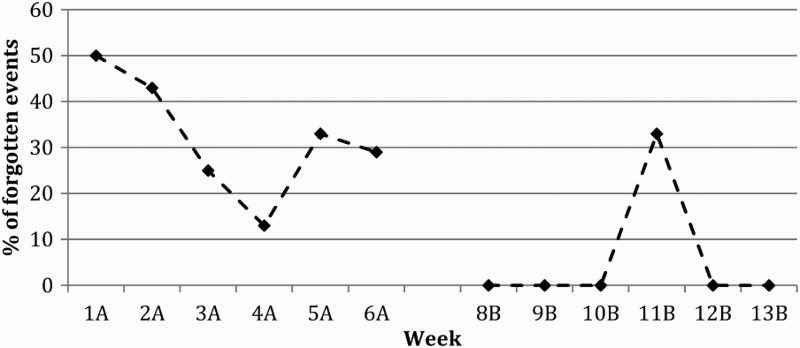
Target events forgotten by JA. A = Baseline Phase, B = Intervention Phase, n.b. Week 7 = trial week.

**Figure 2.**
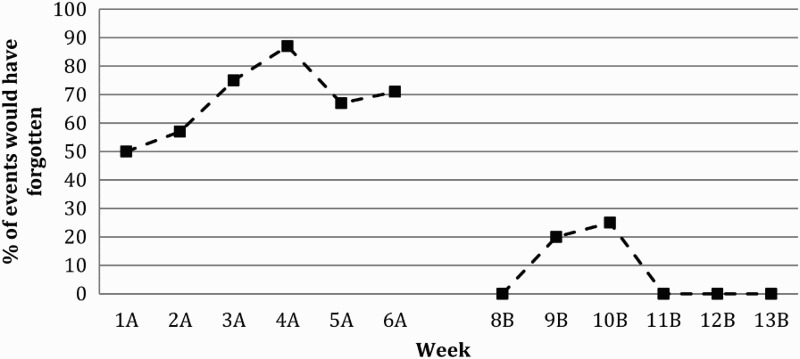
Target events JA would have forgotten if not reminded. A = Baseline Phase, B = Intervention Phase, n.b. Week 7 = trial week.

The total number of target events was 38 in baseline and 22 in the intervention phase; the total number of control events was 42 (including all control events) in the baseline and 35 in the intervention. Tate et al. (2008) suggest that the effectiveness of a treatment should be demonstrated both statistically and visually for single case experimental design (SCED) studies. NAP analysis was therefore utilised. In order to calculate NAP, all target behaviours (i.e., all appointments, days attending the rehabilitation service and days attending the Mosque) were collated to create a total events score for each week. The number of times these events were forgotten was then calculated as a percentage of total events. NAP analysis was used to determine performance change between baseline (phase A) and intervention (phase B) which is shown in the plot of events in Figure 1. NAP analysis revealed there was a 90% improvement in performance between baseline and intervention for the number of target events forgotten ­– Total non-overlap/Total possible pairs×100 = NAP% (32.5/36 = 0.90×100 = 90%). There was also a 100% change in performance for target events that JA would have forgotten if he had not been reminded – Total non-overlap/Total possible pairs×100 = NAP% (36/36 = 1×100 = 100%) as shown in Figure 2. At week 11 during the intervention (Phase B) there is a sudden increase in forgetting events to 33% (see Figure 1) because JA's phone was mislaid and he was not able to receive any text message reminders. Once it was found, JA did not forget any other events for the remainder of the intervention phase.

**Figure 3.**
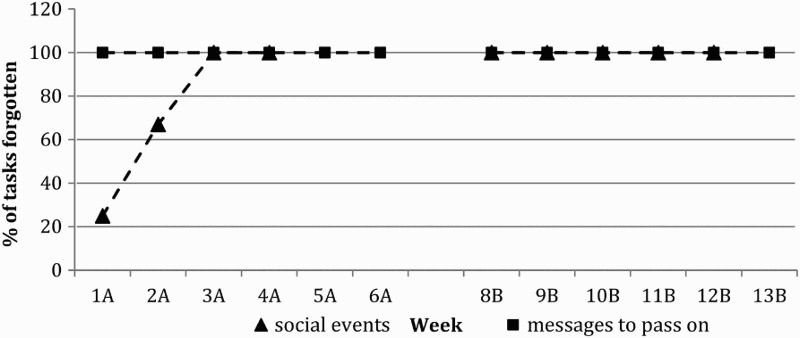
Percentage of control events forgotten. A = Baseline Phase, B = Intervention Phase, n.b. Week 7 = trial week.

**Figure 4.**
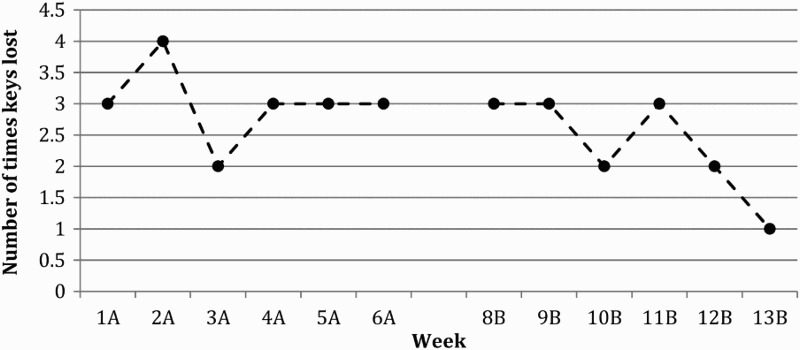
Control task: Number of times JA lost his keys each week. A = Baseline Phase, B = Intervention Phase, n.b. Week 7 = trial week.

NAP analysis could not be conducted on the control condition “Forgetting to pass on messages to partner” as this remained constant throughout both the baseline and intervention phase suggesting no change in the behaviour (Figure 3). “Social events forgotten” could not be analysed as in weeks 5, 6 and 13 no social events occurred (Figure 3). However, visual analysis shows that forgetting social events increased over the baseline period and remained constant during the intervention phase. The total number of times JA lost his keys decreased between the baseline and intervention phases (Figure 4). NAP analysis revealed a 72% change in performance. During the initial baseline phase JA started to keep his phone in a specific place and spontaneously instigated the same strategy in order to remember where his keys were, which accounts for the decrease in the number of times JA lost them (Figure 4).

Analysis of the BMQ suggested that JA's personal control and treatment control beliefs increased for the better post-intervention (Table 2). Inappropriate beliefs about memory aids, e.g., “It's better to try to rely on my own memory than use memory aids”, decreased as did threat appraisals, e.g., “Using a memory aid would make me feel like I need help”. Lifestyle beliefs, e.g., “Using a diary just doesn't fit my lifestyle”, increased marginally. EMQ-r scores also reduced suggesting that JA no longer experienced forgetting as frequently as he had prior to the intervention.

**TABLE 2:** Total BMQ sub-scale scores and EMQ-r pre- and post-intervention

	*BMQ personal control beliefs (Max score 24)*	*BMQ treatment control beliefs (Max score 24)*	*BMQ inappropriate beliefs (Max score 24)*	*BMQ threat appraisal (Max score 36)*	*BMQ lifestyle fit (Max score 20)*	*EMQ-r total score (Max score 52)*
Pre	16	22	18	28	15	52
Post	22	24	14	21	16	35

## DISCUSSION

This single case multiple baseline study across behaviours, demonstrates that after careful consideration of the client's beliefs and requirements it is possible to introduce an effective memory strategy to a client who is not predisposed towards using memory aids.

Baseline data confirmed that JA often forgot to carry out target behaviours or only carried them out if his partner reminded him. Both visual and statistical analysis suggested an improvement in target behaviours over baseline after Google Calendar was introduced. Improvement was not due to spontaneous recovery as there was no change in two of the three control behaviours. JA showed improvement on the third control behaviour because he spontaneously applied a compensatory strategy. Subjective complaints also reduced as can be seen by JA's score on the EMQ.

JA reported that the memory aid had “helped him 100%” and no one knew that the text messages he was receiving were memory prompts. JA noted that his anxiety about using a memory aid had reduced and it was something he now “felt happy with”. This was also reflected in some changes on the BMQ which suggested that he felt more in control of his memory problems and his inappropriate beliefs about memory and memory aids decreased. He also had fewer social concerns about using a memory aid.

JA's partner reported that implementing Google Calendar resulted in him becoming less reliant on her and reduced her stress levels. Although no formal follow-up data were collected, it was noted that two months after the study had finished, JA attended a review meeting at the rehabilitation centre without the need for any prompts from the rehabilitation service or his partner. This would not have been achievable prior to the intervention. At the meeting JA stated that he continued to use the Google Calendar and he will continue to use it to help him remember important events and appointments.

This study adds to the growing body of literature suggesting the effectiveness of electronic memory aids for people with prospective memory difficulties (Morris & Reinson, 2010). It also supports the efficacy of alert reminders for people with memory difficulties (e.g., Evans et al., 1998; Wilson et al., 1999; 2001; Wilson, Evans, Emslie, & Malinek, 1997).

In today's society mobile phones are widely used and accepted as an integral part of everyday life, which makes Google Calendar an attractive option as a memory aid. With over four billion mobile phone users worldwide (Electronics Take Back Coalition, 2010) the majority of people who have had an ABI are likely to be familiar with this technology premorbidly. The discreteness that is offered by Google Calendar reduces any potential embarrassment about using aids, since receiving an SMS message on a mobile is a common everyday phenomenon.

The study confirms that an aid should be in keeping with a particular individual's lifestyle, convey their desired self-image and fit their value systems (Baldwin et al., 2011; Bender Pape, Kim & Weiner, 2002). Cultural implications may be important as brain injury may incite feelings of individual and familial shame (Simpson, Mohr, & Redman, 2000; Watanabe, Shiel, McLellan, Kurihara, & Hayashi, 2001). The mechanism of injury may also be important, e.g., for JA, the fact that he experienced an assault may have led to a general mistrust of others and influenced his perception of negative social evaluation (Riley, Brennan, & Powell, 2004).

It has been highlighted that those with executive functioning difficulties can struggle to use external memory aids effectively (Wilson & Watson, 1996; McDonald et al., 2011). However, JA benefited from the use of Google Calendar despite presenting with executive deficits. One explanation may be that JA utilised five alerts for each event and the repetitive nature may have circumvented any propensity to disregard the prompt. It is not known how many alerts per event were used in the study by McDonald et al. (2011) but this could be an area for further research, together with an exploration of which aspects of smartphone use present a challenge to those with executive problems. An additional reason why JA benefited from Google Calendar may be that JA was motivated to complete the tasks which were specifically chosen by JA and his wife as they were important to him. McDonald et al. (2011) highlight that one participant in their study did not successfully complete tasks because the tasks were not personally meaningful. Even in experimental situations, therefore, it may be important for tasks to be meaningful for the individual, although this does present methodological challenges as outlined below.

Although this study shows promising results for the efficacy of Google Calendar as a prospective memory aid there are some design limitations which need to be taken into consideration. Choosing tasks that were meaningful for JA meant that intervention and baseline tasks differed in their nature and frequency. Whilst the three intervention tasks placed demands on prospective memory, only two of the control tasks did so. Losing keys is a retrospective memory task and is not amenable to the intervention of text message alerts. However, in everyday life, prospective memory does depend to some extent on retrospective memory in terms of recalling what has to be done when it is cued. Furthermore, the two types of memory systems are likely to share common resources as implied in a study comparing different factor structures for prospective and retrospective memory (Crawford Smith, Maylor, Della Sala, & Logie, 2003). It was felt, therefore, that it could be an ancillary control measure of spontaneous recovery in memory functioning. Passing on messages is amenable to text message alerts and in fact had been partially implemented during the training week when JA was successfully prompted by three message alerts that were sent over a 1 hour period between 7 p.m. and 8 p.m. to send the researcher details about any upcoming appointments. Thus, a message alert each evening which stated “pass on messages from friends” could remind JA to talk to his wife about anything he had to impart. Upon completion of the study JA stated he would store any information he had to pass onto his wife on his phone as this would be discrete and look like he was writing a text message. He would then be able to retrieve this information following the prompt by the message alert.

Unfortunately, it was not possible to control frequency of events in either the intervention or control task as this was a naturalistic study. Similarly, types of event had to be combined in order to derive sufficient meaningful data. This is a limitation of the current study but it was felt that the benefits of ecological validity compensated for this methodological weakness. Research on the use of PDAs and smartphones as electronic memory aids highlights their continuing value (Savage & Svoboda, 2013), future research may therefore wish to assess whether users of Google Calendar are able to maintain long-term use and the benefits of the calendar system. It should also be noted that in the current study, JA was only monitored on his ability to respond to events and not on his ability to enter events correctly. As demonstrated by Savage and Svoboda (2013) with their client RR, incorrect entries can also be problematical and it is possible that some clients may not have the cognitive ability to do both. Therefore, evaluating both event entry and response would give a fuller picture of the feasibility of Google Calendar.

A limitation of this particular aid, and specifically the use of any electronic aids that utilise a mobile phone network, is that we are unable to control or rectify any difficulties that may occur to the signal systems operated by these networks. For example, JA reported that one week the local mobile phone mast was vandalised and so he forgot a number of tasks. A second limitation is that technology and services are continually developed and updated as a result of growing consumer demand, thus requiring people with memory difficulties to learn new processes. Such changes to Google Calendar occurred during this study when additional functions were added. It should also be noted that those who have utilised electronic devices following ABI have struggled to transfer skills from one device to another (McDonald et al., 2011; Savage & Svoboda., 2013). This needs to be taken into consideration when selecting electronic devices as memory aids for people following ABI.

Nevertheless, affordable, off-the-shelf technology is now available for people with memory problems and so it is no longer necessary to adapt devices, which has been a major limitation in the past (Gillette & DePompei, 2008; Kim et al., 2000; Van den Broek, Downes, Johnson, Days, & Hilton, 2000; Wade & Troy, 2001; Wilson et al., 1997; Wright, Rogers, Hall, Wilson, Evans, & Emslie, 2001; Wright, Rogers, Hall, Wilson, Evans, Emslie, & Bartram, 2001). As mobile technology develops, there has been a rapid transition from standard mobiles to smartphones, with 51% of the UK adult population now owning a smartphone (Ofcom, 2013). Future studies could consider teaching individuals how to access Google Calendar on their smartphone or use email alert reminders which allow for even more information to be recorded about the event. Smartphones may also be used to capture film or photos throughout the day to aid retrospective memory and this may be more socially acceptable than a system such as SenseCam (Hodges et al., 2006). Applications are constantly becoming available for smartphones and it may be possible to develop, for example, an application that enables people with language difficulties to access pictorial calendars. This technology therefore offers a multitude of functions as well as design aesthetics that have a broader appeal.
